# Trichosanthin inhibits cervical cancer by regulating oxidative stress-induced apoptosis

**DOI:** 10.1080/21655979.2021.1930335

**Published:** 2021-07-07

**Authors:** Chenglu Zhu, Cuilan Zhang, Xiaoming Cui, Jing Wu, Zhizhu Cui, Xiaojuan Shen

**Affiliations:** Department of Gynaecology and Obstetrics, Dongtai Hospital of Traditional Chinese Medicine, Dongtai, Yancheng City, Jiangsu Province, 224200, China

**Keywords:** Trichosanthin, cervical cancer, cell proliferation, apoptosis, oxidative stress

## Abstract

Based on many studies, trichosanthin (TCS) has an antiviral effect that regulates immune response, and targets cancer cells to exert broad-spectrum anti-tumor pharmacological activities. It is speculated that TCS may be a potential natural active drug for preventing as well as treating cervical cancer. But the clearer impact along with underlying TCS mechanism on cervical cancer are still unclear. The purpose of this study is to investigate the function and potential mechanism of TCS in cervical cancer. We measured the viability of cervical cancer cell lines (HeLa & caski cells) using CCK-8 analysis, detected cell proliferation efficiency through Ki-67 staining, analyzed cell apoptosis rate via flow cytometry as well as annexin V-FITC/PI double staining, performed apoptosis-related protein expression through western blotting, evaluated cell migration along with invasion by wound as well as transwell assays, carried out MMP via JC-1 and Rh123 fluorescent probes, as well as detected intracellular ATP and ROS levels by flow cytometry, respectively, to evaluate the effects of TCS. We found that TCS inhibited viability along with proliferation, induced apoptosis, as well as inhibited HeLa & caski cell migration along with invasion in a time- and dose-dependent manner. Additionally, TCS also reduced MMP, and the production of adenosine triphosphate, as well as induced the increase of intracellular reactive oxygen species in cancer cell lines. In accordance with the present studies, TCS inhibits HeLa & caski cell proliferation along with migration but promotes their apoptosis, which may be mediated by regulating oxidative stress.

## Graphical Abstract

**Figure:**
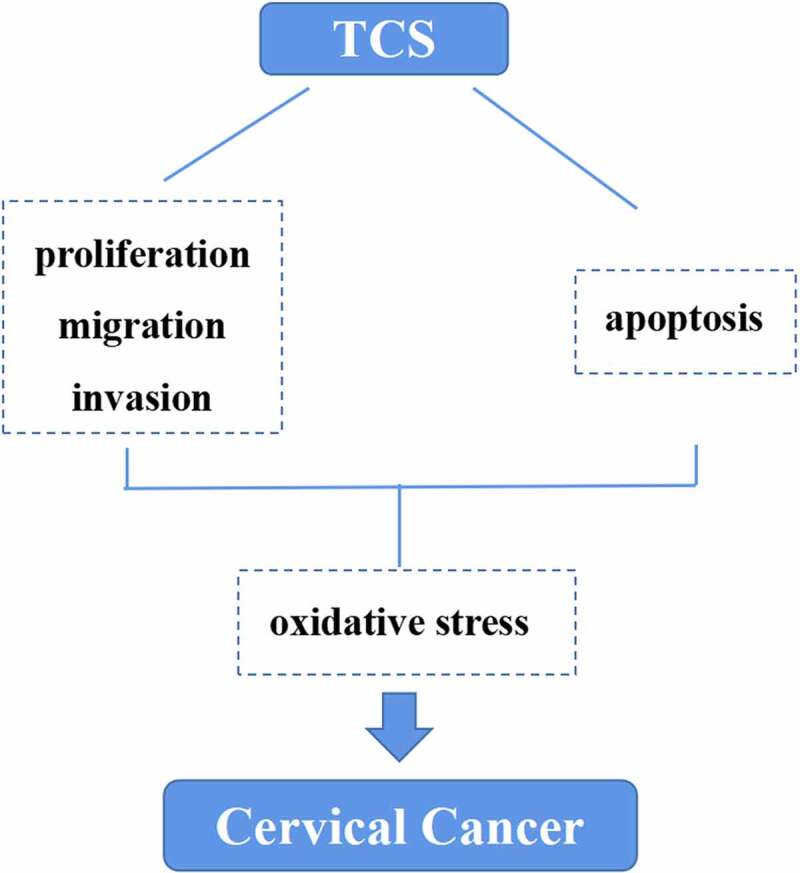

## 1. Introduction

Cervical cancer, one of the most universal as well as fatal gynecological malignancies among women all over the world, results in a very large economic and medical burden worldwide [1–4]. It is estimated that by 2040, the annual number of deaths related to cervical cancer is estimated to reach 460,000, and its mortality rate is second only to breast cancer [5,6]. Treatment strategies for cervical cancer usually include surgery, chemotherapy and radiation therapy, but they are not always completely effective [7–9]. The first-line treatment for cervical cancer is a variety of effective chemical drugs, such as paclitaxel, cisplatin, fluorouracil, cyclophosphamide, and topotecan bevacizumab. But patients usually show severe side effects as well as drug resistance after taking the drugs, leading to tumor recurrence and its further development [10]. Therefore, it is very important to improve the latest understanding of the biological characteristics along with the mechanisms of cervical cancer cells and to develop effectively new and chemotherapeutic drugs are vital for preventing and treating cervical cancer.

TCS is an effective active ingredient extracted from the roots of a common Chinese herbal medicine Trichosanthes kirilowii [11,12]. It features a wide range of pharmacological activities, like immune regulation, antiviral and anti-HIV effects [13–16]. TCS shows strong cytotoxicity in various tumor cell lines, without damage to physiological activity of normal cell lines [17]. In addition, TCS also exhibits anti-tumor activity, like anti-tumor cell proliferation, tumor cell apoptosis induction and other molecular mechanisms [18], so it is speculated that TCS may be a promising targeted drug for preventing and treating cervical cancer [19,20]. But the anti-tumor mechanism of TCS against cervical cancer remains to be further studied.

In this work, we aim to explore the impact of TCS on cervical cancer cell line proliferation, migration along with apoptosis *in vitro*. Additionally, we also explored whether the oxidative stress pathway mediates the effect of TCS on cervical cancer. This work provides an experimental basis for further research on the mechanism of TCS in preventing and treating cervical cancer, and indicates that TCS may be a potential anti-tumor drug for treating cervical carcinoma.

## 2. Materials and methods

### 2.1. Cell culture and reagents

Two kinds of human cervical cancer cell lines (HeLa and caski cell) were purchased from ATCC (Maryland, USA). According to relative operating requirements, cells were cultured in Dulbecco’s modified Eagle’s medium (Gibco) containing 10% FBS (Gibco), 100 U/mL penicillin as well as 100 μg/mL streptomycin in a 5% CO_2_ incubator at 37°C. TCS was purchased from Shanghai Jinshan Pharmaceutical Co., Ltd. as well as melt it in DMSO during the experiment.

### 2.2. Cell viability assay

Based on the manuscript instructions, we applied CCK‑8 (Dojindo) to detect cervical cancer cell line viability, seeded HeLa & caski cells in a 96-well plate at a density of 5 × 10^3^ cells/well, as well as cultured them overnight. Then, TCS with different concentrations (0, 5, 10, 20, 40 and 80 μg/mL) were added to each well in a dose-dependent manner. After incubating for 24, 48, and 72 hours in a time-dependent manner, we removed upper medium, added CCK-8 working reagent (10 μL) to each well along with incubated it for 4 h at 37°C. Later our members applied a SpectraMax M5 plate reader (Molecular Devices LLC) to detect the absorbance value at 450 nm.

### 2.3. Cell morphology

HeLa & caski cells were, respectively, plated in 6-well plates to logarithmic growth phase. Then, cells were treated with TCS of different concentrations (10, 20 and 40 μg/mL), according to the previous results [20]. The inverted microscope (Olympus Corporation, Japan) was used to photograph the cells to observe the morphological changes after 24-hour and 48-hour TCS treatment.

### 2.4. Ki-67 staining

Cervical cancer cell lines (HeLa & caski cells) were treated with TCS (10, 20 and 40 μg/mL) for 48 h, later fixing them in 4% paraformaldehyde. The HeLa & caski cells were stained with anti-Ki-67 antibody (cat.no. ab15580, Abcam) overnight at 4°C, after which we added Goat Anti-Rabbit IgG secondary antibody (cat.no. ab205718, Abcam) to combine with primary antibody. A confocal laser scanning microscope (Olympus Corporation) was used to take pictures of the cells to detect the morphologic changes in the cell nucleus.

### 2.5. Flow cytometry

In accordance with instructions from manufacturer, after TCS treatment, we applied Annexin V-FITC/PI apoptosis detection kit (Sigma‑Aldrich) to determine HeLa & caski cell apoptosis. Specifically, HeLa & caski cells were seeded in 6-well plates (5 × 10^5^/well) as well as handled them with TCS (10, 20 and 40 μg/mL) for 48 h. Then, cells were collected and resuspended in 195 μL of binding buffer, and 5 μL of Annexin V‑FITC and 10 μL propidium iodide (PI) working solution were added for reaction, following incubation at room temperature for 20 min in darkness. Cell apoptosis were detected by flow cytometry (BD FACS Calibur).

### 2.6. Cell migration assay

The wound healing assay was used to measure the impact of TCS on HeLa & caski cell migration. Specifically, HeLa & caski cells grew on the culture plates to reached 80% confluence. Later we used a sterile 10 μL pipette tip to draw a straight line on the cell surface. Later, our members treated cells with TCS (10, 20 and 40 μg/mL) for 48 h. The width of the wound was photographed after 48 h under the microscope (Olympus Corporation, Japan).

### 2.7. Cell invasion assay

The transwell assay was used to assess the impact of TCS on HeLa & caski cell invasion. Specifically, we seeded HeLa & caski cells (1 × 10^5^/well) into the upper chamber of a transwell insert as well as added a medium containing 20% FBS to the lower one. Then treated cells with TCS (10, 20 and 40 μg/mL) and cultured them for 48 h. We stained the cells invading into outer surface of transwell insert with 0.5% crystal violet working solution, calculated along with analyzed them with an inverted microscope (Olympus Corporation, Japan).

### 2.8. Western blotting

We treated HeLa & caski cells with TCS (10, 20 and 40 μg/mL) for 48 h then lysed with RIPA buffer (Sigma-Aldrich, USA) to collect total protein. In accordance with instructions from manufacturer, the total protein concentration of each sample was detected via BCA kit (KeyGEN Biotech). We loaded equal amount of protein on 10% SDS-PAGE (Bio-Rad), later transferring it to PVDF films, which we blocked for 1.5 h at room temperature with 5% nonfat milk, and later incubated it with primary antibody overnight at 4°C. Later, we incubated the films with the horseradish peroxidase-conjugated secondary antibody (Anti-rabbit IgG, cat.no. #7074, Cell Signaling Technology; Anti-Mouse IgG H&L (HRP), cat.no. ab205719, Abcam) at room temperature for 2 h, as well as quantified the gray value of the bands through ECL detection system (Amersham Life Science).

### 2.9. Determining MMP

Decreased MMP signals the apoptosis on the early stage [21]. With aim to assess the impact of TCS treatment (10, 20 and 40 μg/mL) on MMP of HeLa & caski cell, we conducted JC-1 staining. Simply, we placed HeLa & caski cells into 12-well plates. After handling them with TCS for 48 hours and washing them with PBS once, we added 1 mL prepared JC-1 staining working solution (Beyotime), with which we incubated the cells at 37°C for 20 min. Later, the cells were washed twice via pre-cooled 1× JC-1 staining buffer, observed as well as photographed them under inverted fluorescence microscopes (Olympus Corporation, Japan). Red and green fluorescence represented changes in MMP and cell state, respectively. The relative ratio of red-green fluorescence to is universally applied to measure the proportion of cells with mitochondrial depolarization.

The cationic working dye Rhodamine 123 (Beyotime) was also used to detect MMP. In short, we pretreated HeLa & caski cells with TCS (10, 20 and 40 μg/mL) at 37°C for 48 h, later incubating them with 2 µM rhodamine 123 at 37°C in darkness for 30 min. Fluorescence intensity was detected through Flow Sight flow cytometer (Amnis Corporation) and the data were analyzed through IDEAS v 6.1.

### 2.10 Measurement of intracellular ATP and ROS levels

Based on the manufacturer’s protocol, we applied a chemiluminescence ATP analysis kit (Beyotime) to measure ATP production. In short, HeLa & caski cells (1 × 10^5^ cells/well) were seeded into 12-well plates and cultured them overnight. After treating them with 10, 20 and 40 μg/mL TCS for 48 h, respectively, we removed the cell culture medium, as well as added the ATP detection working solution to incubate them together at room temperature for 3 min. Later, our members added 20 µL sample to the wells, and calculated ATP concentration in accordance with standard curve.

Aiming to evaluate the impact of TCS, we applied the ROS sensitive probe 2′,7′-dichlorodihydrofluorescein diacetate (H_2_DCFDA) to detect ROS level in cervical cancer cells. In simple terms, we treated HeLa & caski cells with indicated TCS concentrations for 48 h, and then incubated them with H_2_DCFDA in darkness for 30 min at 37°C. Finally, the fluorescence intensity of cells was measured via the Flow Sight flow cytometer (Amnis).

### 2.11 Statistical analysis

Our work expressed the findings as mean ± standard deviation (SD) and performed statistical analysis through SPSS version 21.0 (Inc.). We analyzed the difference among groups through one-way analysis of variance (ANOVA) before Tukey′s post-hoc test. *P* < 0.05 mean statistical significance.

## 3. Results

In this study, we hypothesized that TCS exerts an anti-tumor effect, which may be mediated by regulating oxidative stress. First, we evaluated the effect of TCS on the cell viability, proliferation rate, apoptosis rate, migration and invasion of cervical cancer cell lines (HeLa and caski cells). In order to study the molecular mechanism, we tested the expression of apoptosis-related proteins. In addition, we verified the mediating role of oxidative stress.

### 3.1. TCS inhibited HeLa & caski cell viability in a time- and dose-dependent way

**Figure 1.**
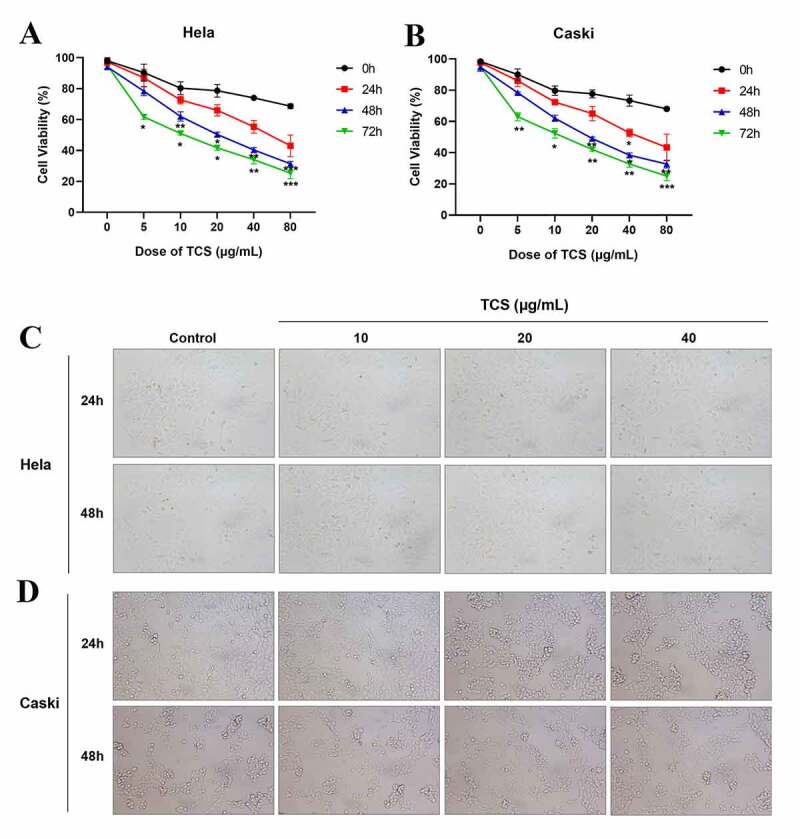
**TCS inhibited HeLa & caski cell viability in a time- and dose- dependent way**. HeLa & caski cells receiving 0, 5, 10, 20, 40 and 80 μg/mL TCS in a dose-dependent manner for 24, 48 and 72 h; (a) HeLa cell viability was evaluated via CCK-8 assay; (b) Caski cell viability was evaluated by CCK-8 assay; (c-d) Morphologic changes in HeLa (c) & caski cells (d) were observed under the ordinary optical microscope (Microscopic magnification 100×); Data were analyzed through one-way ANOVA as well as presented as mean ± SD. *p < 0.05, **p < 0.01, ***p < 0.001, in comparison with control group. TCS, Trichosanthin

With aim to determine the impact of TCS treatment on cervical carcinoma cell viability, we cultured HeLa & caski cells with 0, 5, 10, 20, 40 and 80 μg/mL TCS in a dose-dependent manner for 24, 48 and 72 h, as well as determined cell viability via the CCK-8. In comparison with the control group, TCS treatment signally inhibited HeLa (Figure 1a) & caski cell viability (Figure 1b) in a time- and dose-dependent way. Observed under the microscope, changes in cell morphology verified the inhibitory effect of TCS treatment on HeLa & caski cells, too. Our team observed cytoplasmic contraction, membrane blistering as well as nuclear condensation in HeLa & caski cells (Figure 1c-d). Based on those findings above, treating with 10, 20 and 40 μg/mL TCS for 48 h had significant effect on cell viability of cervical cancer cells.

### 3.2. TCS inhibited proliferation and induced apoptosis of HeLa & caski cells

**Figure 2.**
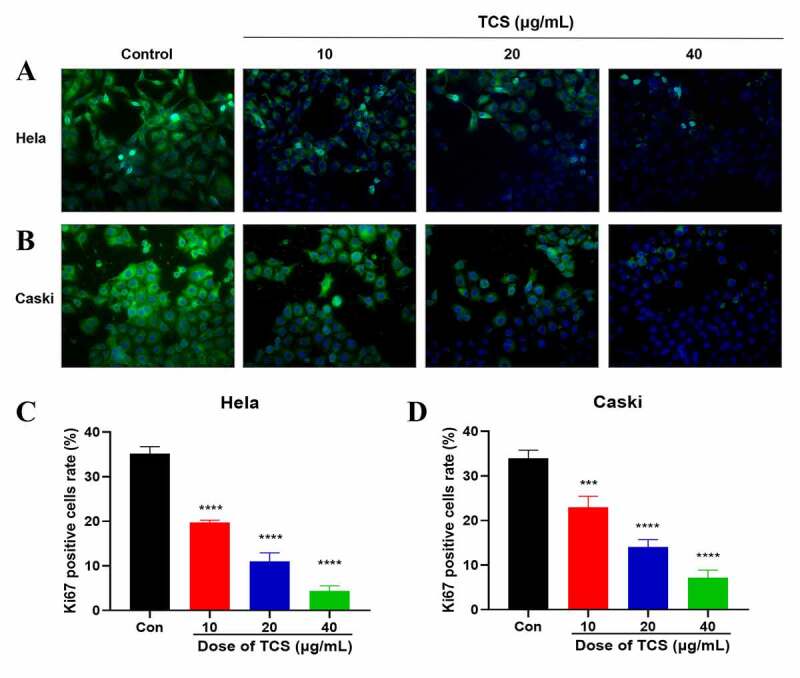
**TCS inhibited HeLa & caski cell proliferation**. (a-b) Representative images of Ki67 staining of HeLa (a) & caski cells; (b) (Microscopic magnification: 200×) Green fluorescence indicates Ki67, Blue fluorescence indicates DAPI; (c-d) HeLa & caski cell proliferation determined via Ki67 staining; TCS, Trichosanthin

**Figure 3.**
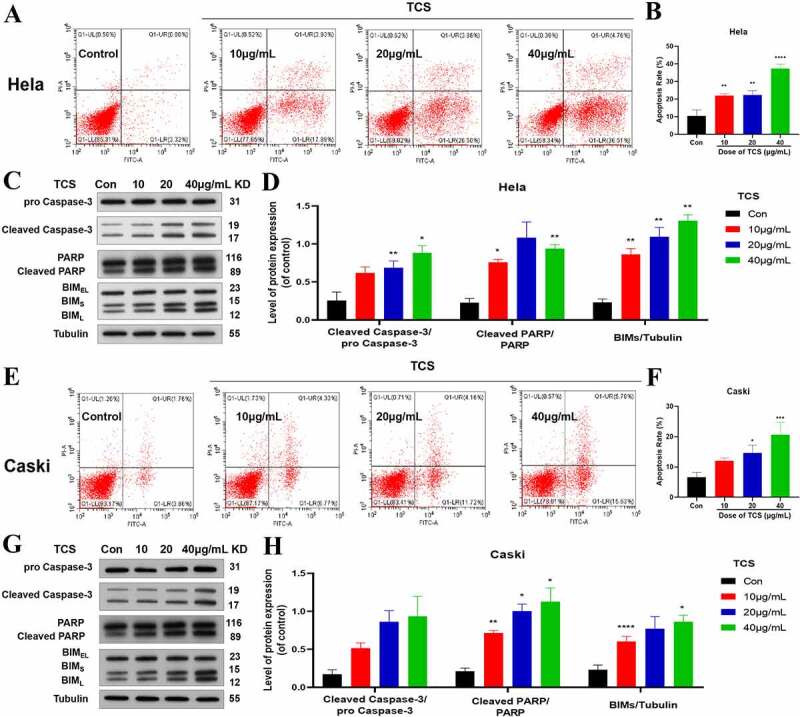
**TCS-induced apoptosis in HeLa & caski cells**. (a-b) Flow cytometric analysis on the cell apoptosis rate in HeLa cells; (c-d) Western blotting for detecting cell apoptosis markers (Caspase-3, PARP, and BIMs) in HeLa cells; (e-f) Flow cytometric analysis on the cell apoptosis rate in caski cells; (g-h) Western blotting for detecting cell apoptosis markers (Caspase-3, PARP, and BIMs) in caski cells. Data were analyzed through one-way ANOVA along with expressed as mean ± SD. *p < 0.05, **p < 0.01, ****p < 0.0001, in comparison with control group. TCS, Trichosanthin

Aiming to study the impact of TCS treatment on cervical cancer cell proliferation, we cultured the HeLa & caski cells with 10, 20 and 40 μg/mL TCS for 48 h, respectively, later staining with Ki-67 staining. Based on the findings of Ki67 staining, in comparison with the control group, TCS treatment resulted in a significant decline in the number of Ki67-positive cell in HeLa (Figure 2a) & caski cells (Figure 2b), indicating that TCS treatment had a proliferation inhibitory effect on cervical cancer cells. Furthermore, we examined whether cell apoptosis was related to cell proliferation inhibition. Based on the findings of flow cytometry analysis, TCS treatment visually elevated apoptotic percentage in HeLa (Figure 3a-b) & caski cells (Figure 3e-f), in comparison with the control group. Additional western blotting analysis also clarified the apoptosis induced by TCS treatment in both HeLa (Figure 3c-d) & caski cells (Figure 3g-h), accompanied with a significant increase in pro-apoptotic proteins, like cleaved-caspase-3, cleaved-PARP, along with BIM in a dose-dependent manner.

### 3.3. TCS inhibited HeLa & caski cell migration and invasion

**Figure 4.**
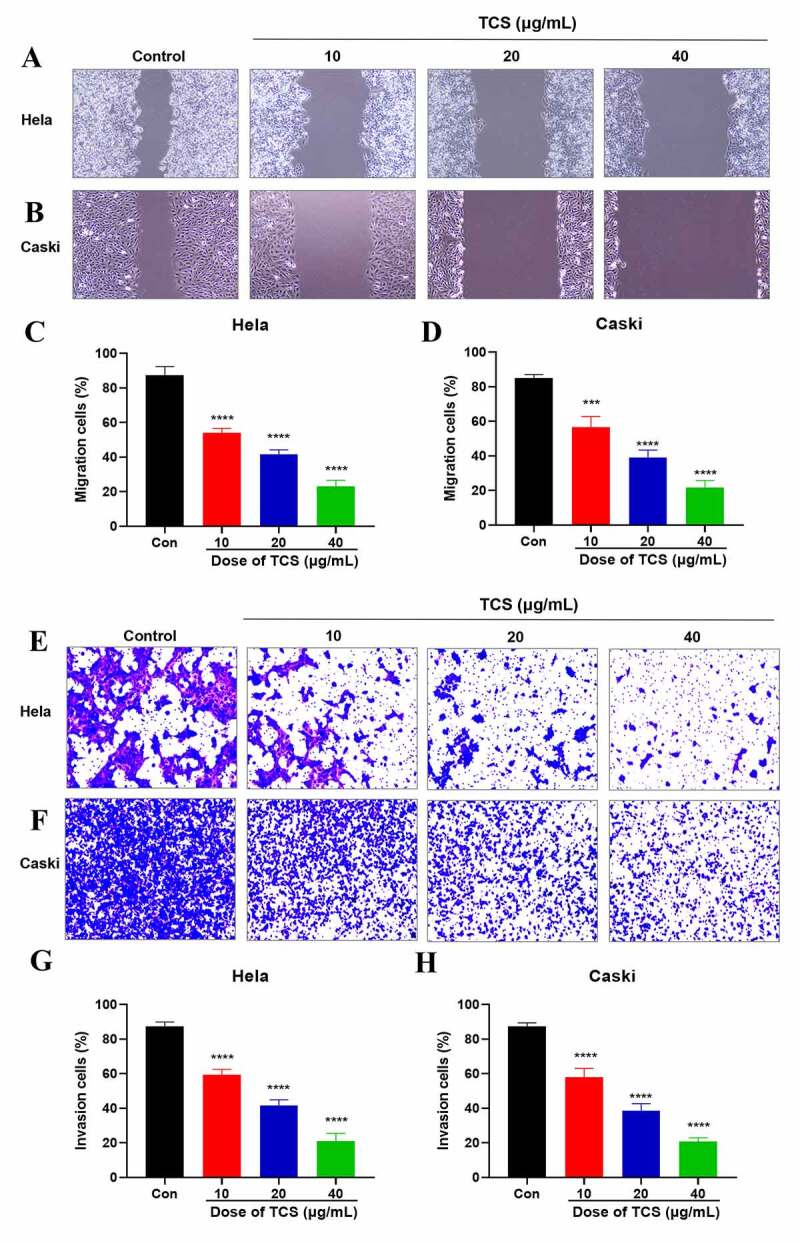
**TCS inhibited HeLa & caski cell migration and invasion**. (a-d) Wound healing assay applied to evaluate the impact of TCS treatment on HeLa (a and c) & caski cell migration; (b and d) (Microscopic magnification 100×). (e-h) Transwell assay applied to assess the impact of TCS treatment on HeLa (e and g) & caski cell invasion (f and h) (Microscopic magnification 200×); TCS, Trichosanthin

In accordance with wound healing assay findings, 10, 20 and 40 μg/mL TCS treatment for 48 h visually inhibited the migration ability of HeLa (Figure 4a) & caski cells (Figure 4b), in comparison with the control group. In addition, we performed transwell assays to assess the impact of TCS on cervical cancer cell invasion. Performing 10, 20 and 40 μg/mL TCS treatment for 48 h visually inhibited HeLa (Figure 4c) & caski cell invasion (Figure 4d), in comparison with the control group (Figure 4c-d).

### 3.4. TCS inhibited mitochondrial dysfunction, decreased ATP production as well as induced ROS in HeLa & caski cells

**Figure 5.**
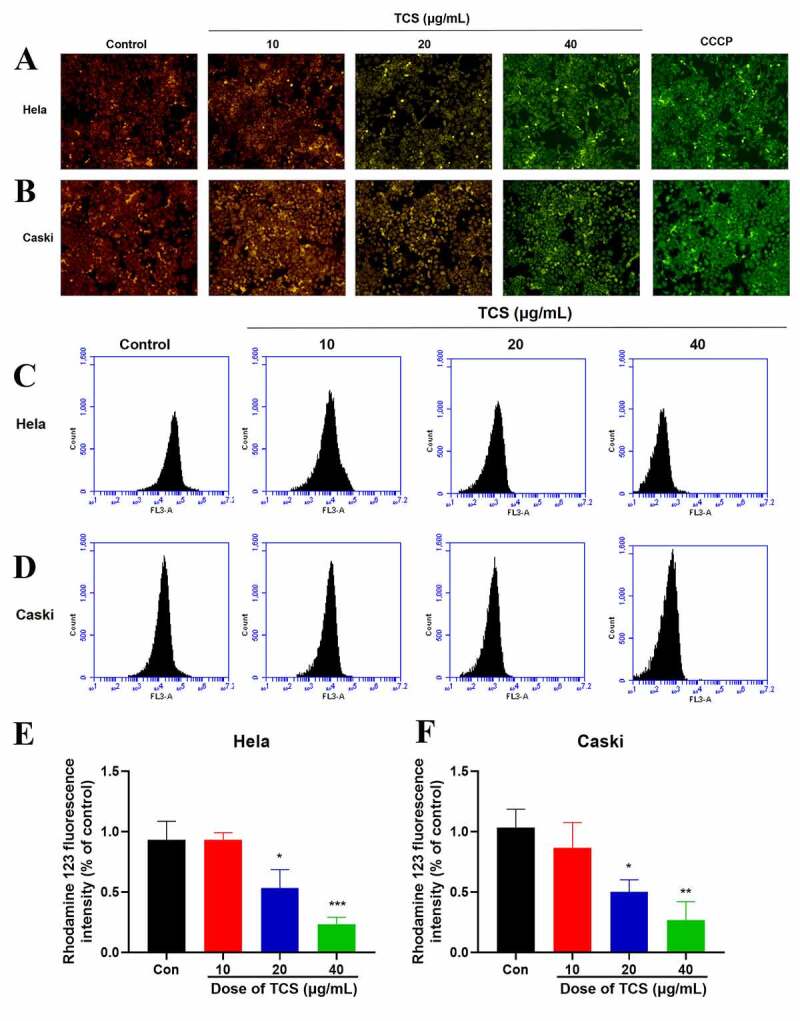
**TCS-induced MMP of cervical cancer cells**. (a-b) Impact of TCS on HeLa (a) & caski cells; (b) MMP was measured by JC-1 staining (Microscopic magnification 200×); (C & F) Impact of TCS on HeLa (C & E) & caski cells (D & F); MMP was measured by rhodamine-123 staining; Data were analyzed via one-way ANOVA, expressed as mean ± SD. *p < 0.05, **p < 0.01, ***p < 0.001, compared with control group. TCS, Trichosanthin

**Figure 6.**
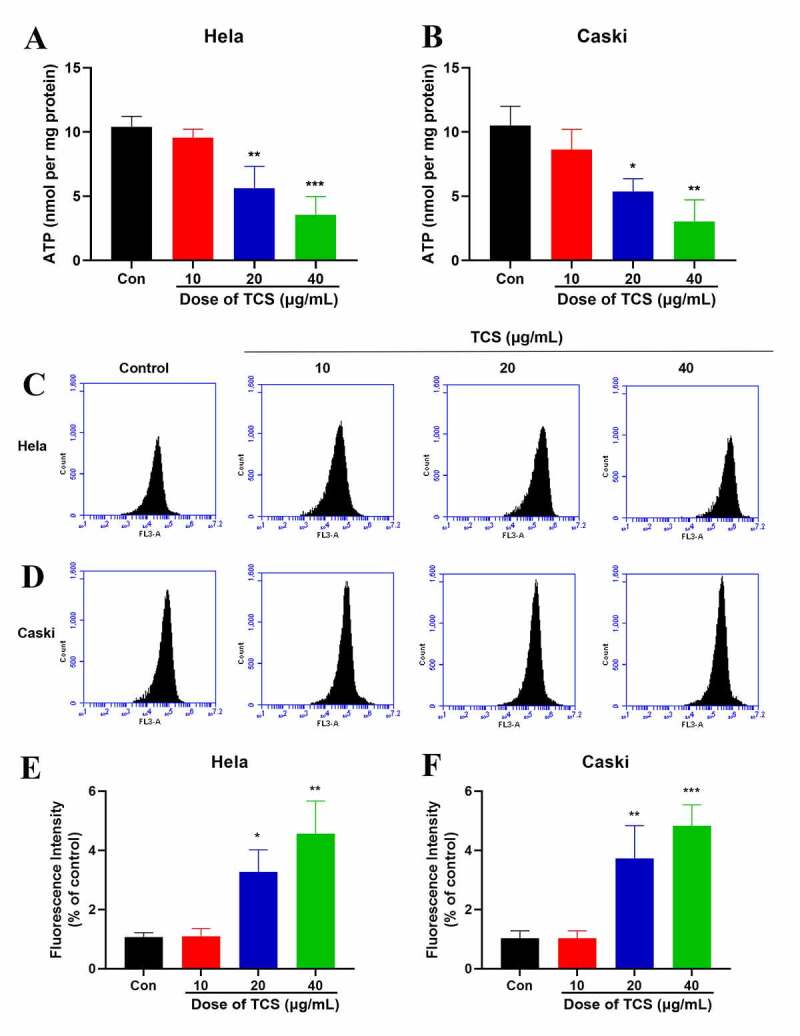
**TCS decreased ATP generation as well as induced ROS of cervical cancer cells**. (a-b) The ATP level of HeLa (a) & caski cells; (b) TCS treatment was measured using a chemiluminescence ATP assay kit; (c-f) ROS level of HeLa (C & E) & caski cells (D & F) treated with TCS was measured. Data were analyzed through one-way ANOVA, and expressed as mean ± SD. *p < 0.05, **p < 0.01, ***p < 0.001, in comparison with control group; TCS, Trichosanthin

The normal function of mitochondria acted pivotally in cell proliferation along with apoptosis [22]. Therefore, we next assessed whether apoptosis induced by TCS was related to mitochondrial dysfunction. We assessed the impact of TCS on MMP, an indicator of mitochondrial functions, through JC-1 as well as rhodamine-123 staining. Based on inverted fluorescence microscopy, cervical cancer cells exposed to TCS revealed increased conversion of JC-1 aggregates (red) to JC-1 monomers (green). After treating with 10 µM CCCP as positive control, the cells revealed obvious green fluorescence, revealing that 10 µM CCCP almost fully induced MMP of HeLa (Figure 5a) & caski cells (Figure 5b). In accordance with flow cytometry results, TCS visually reduced the fluorescence intensity of rhodamine-123 (Figure 5c-f). Given that MMP and ATP production were important indicators of mitochondrial function [23], we evaluated the impact of TCS on the cellular ATP levels. In comparison with control group, TCS reduced cellular ATP levels in HeLa (Figure 6a) & caski cells (Figure 6b). It was speculated that TCS-induced mitochondrial dysfunction may lead to elevated ROS level. Aiming to test this hypothesis, ROS level in HeLa & caski cells receiving TCS treatment were measured. TCS with low concentration imposed no visual impact on cellular ROS, while medium- and high-dose TCS (20 and 40 µM) significantly increased cellular ROS in HeLa (Figure 6c) & caski cells (Figure 6d), in comparison with control group. These findings revealed that TCS inhibited mitochondrial dysfunction in cervical cancer cells, which was manifested via a decrease in MMP and ATP levels and an increase in ROS level.

## 4. Discussion

Cervical cancer is globally among the dominant reasons for cancer-related deaths in females [24–27]. Evidence from clinical and experimental studies has shown that TCS has anti-tumor activity in a variety of anti-cancer treatments, including cervical cancer [16,28,29]. However, so far, the exact effects and mechanisms of anti-tumor activity of TCS in cervical carcinoma is not fully understood. Previous studies have reported that TCS inhibits the proliferation of cervical cancer cells and downregulates STAT-5/C-myc signaling pathway [30]. Here, we demonstrated that TCS treatment was available to reduce cervical cancer cell viability in a time- along with dose-dependent way, thus inhibiting cell proliferation along with promoting cell apoptosis, accompanied with increased caspase-3, PARP and BIM protein levels. In addition, TCS-induced apoptosis is related to mitochondrial oxidative stress.

It is said that TCS exerts anti-tumor activity through inducing cell apoptosis as well as inhibiting cell proliferation [31,32]. Research has shown that TCS could induce Hela cells apoptosis through elevating cytosolic calcium, while inhibiting cAMP/protein kinase C level [33], and Hela cells proliferation through suppressing PKC/MAPK signaling pathway [34]. Based on our work, TCS treatment signally inhibit cell viability, HeLa & caski cells proliferation, consistent with previous studies, and this is also an essential mechanism for many drugs to exert anti-tumor efficacy [35]. Through TCS treatment, the proportion of apoptotic cell was in line with cervical cancer cell proliferation. These findings proved that the inhibitory impact of TCS treatment on the proliferation of HeLa & caski cells were mediated via inducing cell apoptosis. It has been known that caspases are both the initiators and the executors of cell death that initially trigger the cellular apoptosis, among which the caspase-3 is the well-characterized [36]. After being activated by auto-proteolysis, the executor caspase-3 is cleaved off by the initiator caspases, which further promotes the cleavage of specific cellular substrates [37,38]. During this process, several important apoptotic hallmarks were observed, including chromatin condensation, plasma membrane asymmetry and cellular blebbing [38], which eventually induce the typical morphological change of cells [39]. In line with this notion, the elevation of cleaved-caspase-3 accounted for the morphological change of both HeLa & caski cells after TCS administration. Except for caspases, western blotting experiments in our study also demonstrated that treatment of TCS, in a concentration-dependent way, induced cervical cancer cells apoptosis through enhancing cleaved-PARP expression along with BIM.

Mitochondrial dysfunction was a key factor in cell apoptosis and acted as an integrated sensor for various death stimuli in cells. For example, it played a key role in maintaining redox balance [40]. The superoxide produced by the mitochondrial respiratory chain complex I and complex III was the main component of intracellular ROS [41], which interfered with the redox balance [42]. This is a well-known cause of apoptosis induced by oxidative stress [43]]. In this study, we found that TCS treatment resulted in increased ROS levels, loss of ΔΨm, and a sharp drop in ATP levels.

## 5. Conclusions

We found that mediating oxidative stress signaling pathways may be the mechanism by which TCS treatment inhibits the growth of cervical cancer, which highlighting the theoretical basis of cervical cancer treatment. However, due to its strong antigenicity, adverse reactions such as anaphylactic shock may occur during clinical application. Therefore, further research should strengthen the safety research of the effective ingredients of TCS, and provide a basis for the clinical application and resource development and utilization of TCS.

## Research highlights

1. TCS inhibits the vitality of cervical cancer cells and induces apoptosis.

2. TCS reduces ATP production in cervical cancer cells and increases ROS.

3. The anti-cervical cancer effect of TCS is mediated by regulating oxidative stress.
